# Study protocol: a cluster randomised controlled trial of a school based fruit and vegetable intervention – Project Tomato

**DOI:** 10.1186/1472-6963-9-101

**Published:** 2009-06-16

**Authors:** Meaghan S Kitchen, Joan K Ransley, Darren C Greenwood, Graham P Clarke, Mark T Conner, Jennifer Jupp, Janet E Cade

**Affiliations:** 1 Leeds Institute for Genetics, Health and Therapeutics, University of Leeds, Leeds, UK; 2 Department of Geography, University of Leeds, Leeds, UK; 3 Institute of Psychological Sciences, University of Leeds, Leeds, UK; 4 National Foundation for Educational Research, The Mere, Upton Park, Slough, Berkshire, UK

## Abstract

**Background:**

The School Fruit and Vegetable Scheme (SFVS) is an important public health intervention. The aim of this scheme is to provide a free piece of fruit and/or vegetable every day for children in Reception to Year 2. When children are no longer eligible for the scheme (from Year 3) their overall fruit and vegetable consumption decreases back to baseline levels. This proposed study aims to design a flexible multi-component intervention for schools to support the maintenance of fruit and vegetable consumption for Year 3 children who are no longer eligible for the scheme.

**Method:**

This study is a cluster randomised controlled trial of Year 2 classes from 54 primary schools across England. The schools will be randomly allocated into two groups to receive either an active intervention called Project Tomato, to support maintenance of fruit intake in Year 3 children, or a less active intervention (control group), consisting of a 5 A DAY booklet. Children's diets will be analysed using the Child And Diet Evaluation Tool (CADET), and height and weight measurements collected, at baseline (Year 2) and 18 month follow-up (Year 4). The primary outcome will be the ability of the intervention (Project Tomato) to maintain consumption of fruit and vegetable portions compared to the control group.

**Discussion:**

A positive result will identify how fruit and vegetable consumption can be maintained in young children, and will be useful for policies supporting the SFVS. A negative result would be used to inform the research agenda and contribute to redefining future strategies for increasing children's fruit and vegetable consumption.

**Trial registration:**

Medical Research Council Registry code G0501297

## Background

The United Kingdom Government's national 5 A DAY programme forms part of the strategy to raise awareness of the health benefits of fruit and vegetable consumption, and to improve access to fruit and vegetables. One aspect of the 5 A DAY programme is the School Fruit and Vegetable Scheme (SFVS) which provides a free piece of fruit or vegetable to children aged four to six years each school day. The scheme has been rolled out nationally during 2004, and involves distributing approximately 440 million pieces of fruit or vegetable each year to over two million children in 18,000 schools across England. The Department of Health has agreed to continue funding the scheme with a further £77 million investment.

A formal evaluation of the SFVS was carried out by our team in 2004[1]. This evaluation was conducted in schools from two areas – the North East and a matched comparison sample from Yorkshire and Humberside; ninety-eight schools in total. Children were assessed at baseline prior to the introduction of the SFVS and again following introduction of the scheme in the North East with Yorkshire and Humberside acting as comparison schools. Results from this evaluation of the SFVS suggest that most children do not consume enough fruit and vegetables[2]. In particular, the evaluation showed that children significantly increased their fruit and vegetable intake following the introduction of the SFVS by 0.5 portion/day. However, once children move from Year 2 into Year 3 when free fruit is no longer available consumption returns to baseline.

The SFVS is one of the largest public health interventions in Britain in recent years. It is important to assess both the longer term impact on children's diets and the sustainability of the scheme in schools with a focus on social inequalities. It is also important to try to maintain fruit and vegetable consumption once the children enter Year 3 and are no longer eligible for the SFVS.

Interventions, apart from the SFVS, have been shown to increase fruit and vegetable intake in children [3-5]. A review by Ciliska et al[6], on the effectiveness of community interventions for increasing fruit and vegetable consumption in children, stated that the most successful interventions gave clear messages, incorporated multiple reinforcing strategies, involved the family, were more intensive, provided over a longer period of time (i.e. not just one or two contacts) and were based on a theoretical framework

The strategies developed and tested in this intervention will be based upon these successful elements and built on a framework related to maintenance of health behaviour. In contrast to social cognition models that focus on initiation of health behaviour, maintenance of health behaviour theories state the need to understand factors determining maintenance over prolonged periods of time. Five theories that suggest factors important in the decision to initiate behaviour change will form the theoretical basis for development of a model suitable for behaviour maintenance in children. These theories include the role of satisfaction; self-efficacy; social support; self-determination theory; and the relapse prevention model [7-11].

### Aims of the study

• To develop an intervention aimed at maintaining fruit and vegetable consumption in Year 3 children (aged 7–8 years)

• To conduct a randomised controlled trial on the effectiveness of the intervention in maintaining fruit and vegetable intake compared with a minimal intervention.

## Method/Design

### Population

The schools for this study will be randomly selected from a sample of 130 chosen to take part in a national cross-sectional study to describe fruit and vegetable intake in Year 2 children. These schools will be chosen to be representative in terms of ethnicity, deprivation, achievement, and region of England, and will be stratified on ethnicity measured by percentage non-white in each school (split into thirds), deprivation measured by the percentage of free school meals at each school (split into fifths), achievement measured by performance at key stage 1 (split into fifths) and region (North, Midlands and South of England). The inclusion criteria for this sample will be all of schools containing pupils in years 2–4, with a minimum year group size of 15 pupils. The exclusion criteria will be small schools with less than 15 pupils per year group; independent schools, special schools, schools without all three years 2–4, and schools that are or have previously participated in National Foundation for Educational Research (NFER) projects or have participated in previous SFVS projects.

### Sample size

From the nationally representative sample, a sub-set of schools will be recruited into the intervention trial. Cluster randomisation will be used, randomising at the school level, as the intervention will involve whole lesson plans and school-level policies. A sample of 25 children (one Year 2 class) from each school, will give a design effect of approximately 3.4 to take account of the cluster randomisation. To have 90% power to detect a 0.5 portion difference in fruit intake, 500 children per group are required, i.e. about 20 schools in each group. Based on results from our initial evaluation of the SFVS, 75% who completed CADET at baseline also completed the follow up CADET. To allow for this margin of safety, 27 schools per group will be selected in each group, i.e. 54 schools in total. The size of effect the study is powered to detect (one half of a portion of fruit/vegetable) was chosen because it was considered the smallest improvement in intake that was worthwhile detecting with an achievable sized sample, and considering the nature of the intervention.

### Randomisation

To ensure balance on important potential confounders within the trial, block randomisation within strata will be used, stratifying on measures of ethnicity (% non-white) and deprivation (% Free School Meals), both split at the median. Whilst we would like to stratify on variables such as region and achievement, there are not enough schools to give large enough strata to do this. Block randomisation will use block sizes of two because allocation will take place in one go, so there will be no opportunity for prediction of which group the schools will be in.

### Study procedure

Eligible schools will be sent a letter, additional information and a consent form inviting them to take part in the study. Consenting schools will then be contacted to arrange an appropriate day to collect baseline information from the children. A study recruitment form will also be sent to the schools/Year 2 teachers. The teacher will use this form to record which children obtained parental consent and which children were absent on the CADET day. Parental, opt-out consent will be obtained prior to data collection. Baseline dietary information using CADET and anthropometric measures of the children will be collected by the fieldworker in the school.

Following baseline data collection, the randomly selected schools for the trial will be sent a letter informing them whether they are in the active or less intensive intervention. The allocation sequence will be generated by the trial statistician. All schools will be allocated at the same time. Time between notification of allocation and receiving the first intervention material will be kept to a minimum to minimise withdrawals at that stage.

Blinding of the schools to their intervention group will not be possible because of the nature of the intervention. Fieldworkers will be blinded to the allocation of schools to the intervention and control arms of the study. Schools will be followed up 18 months later when the sample of children are in year 4 (aged 8–9 years). They will have further measures of diet using CADET and anthropometric measures taken.

### Study intervention

Using the theoretical framework of maintenance of health behaviour the Project Tomato intervention will employ the following principles – familiarisation, repetition, activities, modelling, and environment (FRAME). Research has shown consistently that the more children are exposed to tasting fruit and vegetables (*familiarisation*) in positive and supportive *environments*, the more willing they are to try them and the more likely they are to like and accept them as a regular component of their diet. Research has also shown that *repeated *exposure to fruit and vegetables encourages children to eat them. Children model their eating behaviour on those around them. This means positive peer and adult role *models *are vital in getting children to accept fruit and vegetables as a regular and likeable part of the diet. Another key finding from research shows that the more children interact with these foods through *activities *such as growing, cooking and tasting, the more likely they are to like and accept them. In some cases, school provides the only opportunity for children to learn about the importance of these foods into their diet [7-11].

The intervention consists of a number of core elements together with some customised elements to meet the needs of each school. The duration of the intervention will be 10 months. The customised elements will be selected and agreed with schools according to their existing cooking or growing facilities, and programmes relating to fruit and vegetables. The final tailored intervention package will be agreed with staff at each school.

### Control Group

An easy to read coloured 5-A Day booklet "Just eat more fruit and veg[12]" will be provided to control schools along with links to relevant websites: one per pupil to send home. At the end of the study the control schools will also be offered access to the full intervention materials if they requested them.

### Outcome measurements

#### Primary outcome

The primary outcome will be the ability of the intervention (Project Tomato) to maintain consumption of fruit and vegetable portions following the exit of children from the SFVS over 18 months compared to the control group.

### Secondary outcomes

#### Diet

Intake of key nutrients including; total energy(MJ/day), fat(g/day), salt(g/day), sugars (g/day), carotene mg/day), and vitamin C (mg/day) will be assessed using the CADET, which has been validated in an ethnically diverse population[13].

#### Behavioural

Children's attitude to fruit and vegetable consumption as measured by a set of ten questions embedded in CADET[14].

#### Anthropometric measures

Weight (kg) measured using standardised procedures and equipment. Body mass index (kg/m^2^) based on weight (as above) and height measured using standardised procedures and equipment following training from the Child Growth Foundation, London.

#### School level measures

Involvement of schools in promoting consumption of fruit and vegetables (number of lessons devoted to learning about fruit and vegetables, school food policy, resources, involvement in national/local initiatives) will be measured using a school questionnaire. Assessment of parents/carers involvement in promoting consumption of fruit and vegetables among pupils will be undertaken using questionnaires.

#### Process measures

Process evaluation data will be collected from intervention schools using both quantitative and qualitative methods. The aim will be to document the implementation of the intervention, as well as to describe and compare processes occurring in the intervention and control schools, at school and participant level.

### Ethical considerations

Ethical approval will be obtained through the University of Leeds research ethics committee. Written informed consent from all schools as well as participants' parents will be sought prior to their enrolment into the study.

### Statistical Analysis

A random intercepts model of child-level outcomes will be used allowing for the hierarchical structure of the data caused by cluster randomisation: child within school. An extra level for the class is unnecessary because only one class is included from each school. MLwiN[15] will be used for this analysis. The fixed effect for the intervention group will be included in the model as a single covariate.

Balance of school and child-level variables between the two intervention groups will be assessed at baseline. This will be carried out with the following variables i) at school level: percentage of children with English as an additional language, percentage of non-white children, percentage of children with free school meals eligibility and percentage defined as having special educational needs ii) and at child level: sex, age and each of the primary and secondary child-level outcomes.

#### Process Measures

Stata version 10.1 and MLwiN will be used to analyse process evaluation variables compared with the fruit and vegetable results from CADET. Process measures and feedback on the intervention will be analysed using appropriate statistical methods[16,17].

#### Children's attitudes

The ten items relating to children's attitudes to fruit and vegetables will be analysed using factor analysis to identify the underlying structure of their responses. Kaiser-Meyer-Olkin and Bartlett's test of sphericity will be used to assess the suitability of the data for factor analysis. Kaiser's criterion and scree plots will be used to determine the number of factors extracted. For each factor, internal consistency will be measured using Cronbach's alpha, as there will be a number of potential outcomes assessed with possibly more than one identified factor, this will be repeated at two time points. Then a multivariate analysis of variance will be performed to determine whether the intervention has a differential effect on children's attitudes over time.

## Discussion

This paper describes a cluster randomised controlled trial to investigate the effectiveness of flexible multi-component intervention designed to support the maintenance of fruit and vegetable consumption for Year 3 children in British primary schools. A positive result would identify methods and knowledge of how to maintain fruit and vegetable intake in young children, and will be useful for policies concerning the SFVS and public health initiatives. A negative result would be used to inform the research agenda and contribute to redefining future strategies for increasing children's fruit and vegetable consumption.

## Abbreviations

SFVS: School Fruit and Vegetable Scheme

## Competing interests

The authors declare that they have no competing interests.

## Authors' contributions

JC conceived the study. JR, DG, GC, and JC were applicants for the funding. MK, JR, DG, MC and JC were involved in designing the study and drafting the protocol. All authors and the TSC read and approved the final protocol.

## Pre-publication history

The pre-publication history for this paper can be accessed here:


